# Supplementary material for: No evidence for association between APOL1 kidney disease risk alleles and Human African Trypanosomiasis in two Ugandan populations
Source: PLoS Negl Trop Dis. 2018 Feb 22;12(2):e0006300. doi: 10.1371/journal.pntd.0006300 (PMC5844566; doi:10.1371/journal.pntd.0006300)
Supplement: S1 Table — (DOCX) [file pntd.0006300.s001.docx]

**S1 Table: Candidate genes included in the study**

| **Gene** | **SNP** | **Location (GRCh37 assembly)** | **Consequence** |
| --- | --- | --- | --- |
| CFH | rs1061170 | 1:196659237 | Missense variant |
| IL10 | rs1800872 | 1:206946407 | Upstream gene variant |
| IL1B | rs1143629 | 2:113593518 | Intron variant |
| IL8 | rs114259658 | 4:74605639 | Upstream gene variant |
| IL8 | rs2227307 | 4:74606669 | Intron variant |
| IL8 | rs2227545 | 4:74608727 | 3 prime UTR variant |
| IL8 | rs13112910 | 4:74609755 | Downstream gene variant |
| IL8 | rs58478511 | 4:74610033 | Downstream gene variant |
| IL8 | rs62312369 | 4:74610397 | Downstream gene variant |
| IL4 | rs2243250 | 5:132009154 | Upstream gene variant |
| IL4 | rs2070874 | 5:132009710 | 5 prime UTR variant |
| IL4 | rs734244 | 5:132010726 | Intron variant |
| IL4 | rs2243255 | 5:132011737 | Intron variant |
| IL4 | rs2243256 | 5:132011753 | Intron variant |
| IL4 | rs2243258 | 5:132012110 | Intron variant |
| IL4 | rs2243261 | 5:132012806 | Intron variant |
| IL4 | rs71889624 | 5:132013430 | Intron variant |
| IL4 | rs2243268 | 5:132013963 | Intron variant |
| IL4 | rs9282745 | 5:132014000 | Intron variant |
| IL4 | rs2243270 | 5:132014109 | Intron variant |
| IL4 | rs2243279 | 5:132016227 | Intron variant |
| IL4 | rs2243282 | 5:132016554 | Intron variant |
| IL4 | rs2243283 | 5:132016593 | Intron variant |
| IL4 | rs2243285 | 5:132016993 | Intron variant |
| IL4 | rs73269366 | 5:132018749 | Downstream gene variant |
| IL12B | rs3212227 | 5:158742950 | UTR variant 3 prime |
| IL12B | rs2546890 | 5:158759900 | Intron variant |
| HLA-A | rs1059563 | 6: 29911928 | Missense variant |
| HLA-G | rs142798055 | 6:29793404 | Upstream gene variant |
| HLA-G | rs1736936 | 6:29794317 | Upstream gene variant |
| HLA-G | rs17875389 | 6:29794484 | Upstream gene variant |
| HLA-G | rs1130355 | 6:29795993 | Synonymous variant |
| HLA-G | rs17875406 | 6:29797448 | Synonymous variant |
| HLA-G | rs1130363 | 6:29797696 | Synonymous variant |
| HLA-G | rs1130363 | 6:29797696 | Synonymous codon |
| HLA-G | rs1632932 | 6:29798039 | Intron variant |
| HLA-G | rs371194629 | 6:29798581 | 3 prime UTR variant |
| HLA-G | rs17179108 | 6:29798642 | 3 prime UTR variant |
| HLA-G | rs9380142 | 6:29798794 | 3 prime UTR variant |
| HLA-G | rs1610696 | 6:29798803 | 3 prime UTR variant |
| HLA-G | rs1233330 | 6:29799103 | Downstream gene variant |
| HLA-G | rs1611139 | 6:29799116 | Downstream gene variant |
| HLA-G | rs2517898 | 6:29799746 | Downstream gene variant |
| HLA-G | rs141206123 | 6:29799849 | Downstream gene variant |
| HLA-G | rs12661041 | 6:29800062 | Downstream gene variant |
| HLA-G | rs2517897 | 6:29800101 | Downstream gene variant |
| HLA-G | rs12662618 | 6:29800211 | Downstream gene variant |
| Intergenic between HLA-G and HLA-A | rs34783406 | 6:29832415 | indel |
| HLA-A | rs1136754 | 6:29911921 | Synonymous variant |
| HLA-A | rs1136754 | 6:29911921 | Synonymous codon |
| HLA-A | rs1059564 | 6:29911930 | Synonymous variant |
| TNFA | rs1800630 | 6:31542476 | Downstream gene variant |
| TNFA | rs1800629 | 6:31543031 | Downstream gene variant |
| IL6 | rs62449495 | 7:22764338 | Upstream gene variant |
| IL6 | rs2069830 | 7:22767137 | Missense variant |
| IL6 | rs2069834 | 7:22767828 | Intron variant |
| IL6 | rs2069837 | 7:22768027 | Intron variant |
| IL6 | rs1474347 | 7:22768124 | Intron variant |
| IL6 | rs2066992 | 7:22768249 | Intron variant |
| IL6 | rs2069842 | 7:22769310 | Intron variant |
| IL6 | rs1548216 | 7:22769773 | Intron variant |
| IL6 | rs2069843 | 7:22769994 | Intron variant |
| IL6 | rs2069845 | 7:22770149 | Intron variant |
| IL6 | rs2069855 | 7:22772624 | Downstream gene variant |
| IL6 | rs1818879 | 7:22772727 | Downstream gene variant |
| IFN-G | rs2069728 | 12:68547784 | Intron variant |
| IFN-G | rs2069723 | 12:68548594 | 3 prime UTR variant |
| IFN-G | rs2069722 | 12:68548953 | 3 prime UTR_variant |
| IFN-G | rs2069720 | 12:68549710 | Intron variant |
| IFN-G | rs2069718 | 12:68550162 | Intron variant |
| IFN-G | rs1861493 | 12:68551196 | Intron variant |
| IFN-G | rs2069713 | 12:68552476 | Intron variant |
| IFN-G | rs2430561 | 12:68552522 | Intron variant |
| IFN-G | rs78554979 | 12:68554636 | Upstream gene variant |
| IFN-G | rs2069705 | 12:68555011 | Upstream gene variant |
| TNX4LB | rs152828 | 16: 72123886 | Intron variant |
| IL4R | rs1801275 | 16:27374400 | Missense variant |
| TXNL4B | rs1424241 | 16:72078907 | Intron variant |
| HP | rs8062041 | 16:72088964 | Intron variant |
| HPR | rs7185840 | 16:72102112 | Intron variant |
| HPR | rs2021171 | 16:72110541 | Missense variant |
| IL12RB1 | rs375947 | 19:18180451 | Missense variant |
| IL12RB1 | rs11575934 | 19:18186618 | Missense variant |
| MIF | rs12483859 | 22:24234807 | Upstream gene variant |
| MIF | rs36086171 | 22:24235455 | Upstream gene variant |
| MIF | rs9282783 | 22:24236359 | 5 prime UTR variant |
| MIF | rs36070976 | 22:24236864 | Intron variant |
| MIF | rs2070766 | 22:24237221 | Splice region variant, intron variant |
| MIF | rs35235644 | 22:24237822 | Downstream gene variant |
| MIF | rs2000466 | 22:24237862 | Downstream gene variant |
| MIF | rs34383331 | 22:24238079 | Downstream gene variant |
| APOL1 | rs136174 | 22:36661536 | Synonymous codon |
| APOL1 | rs73885316 | 22: 36661674 | Missense variant |
| APOL1 | rs136177 | 22:36661842 | Synonymous codon |
| APOL1 | rs73885319 | 22:36661906 | Missense variant |
| APOL1 | rs143830837 | 22:36662042-36662047 | Coding sequence indel |
| APOL1 | rs71785313 | 22: 36662046:36662051 | Coding sequence indel |
