# Supplementary material for: No evidence for association between APOL1 kidney disease risk alleles and Human African Trypanosomiasis in two Ugandan populations
Source: PLoS Negl Trop Dis. 2018 Feb 22;12(2):e0006300. doi: 10.1371/journal.pntd.0006300 (PMC5844566; doi:10.1371/journal.pntd.0006300)
Supplement: S2 Table — (DOCX) [file pntd.0006300.s002.docx]

**S2 Table: Association results of 65 SNPs with Acute HAT**

| **CHR** | **SNP** | **GENE** | **BP** | **A1** | **F_A** | **F_U** | **A2** | **P** | **OR** | **L95** | **U95** | **BONF** | **FDR_BH** | **FST** | **MAF** |
| --- | --- | --- | --- | --- | --- | --- | --- | --- | --- | --- | --- | --- | --- | --- | --- |
| 1 | rs1061170 | CFH | 196659237 | C | 0.4915 | 0.4788 | T | 0.7833 | 1.052 | 0.7333 | 1.51 | 1 | 0.9257 | -0.00405298 | 0.4852 |
| 1 | rs1800872 | IL-10 | 206946407 | T | 0.4068 | 0.4286 | G | 0.6095 | 0.9143 | 0.6346 | 1.317 | 1 | 0.9257 | -0.00354243 | 0.4177 |
| 2 | rs1143629 | IL-1B | 113593518 | A | 0.4118 | 0.4576 | G | 0.3319 | 0.8296 | 0.5767 | 1.193 | 1 | 0.8752 | 0.000171276 | 0.4346 |
| 4 | rs114259658 | IL-8 | 74605639 | A | 0.05042 | 0.04202 | T | 0.6706 | 1.211 | 0.5127 | 2.858 | 1 | 0.9257 | -0.00363002 | 0.04622 |
| 4 | rs13112910 | IL-8 | 74609755 | G | 0.3655 | 0.3782 | A | 0.777 | 0.9475 | 0.6533 | 1.374 | 1 | 0.9257 | -0.0044355 | 0.3718 |
| 4 | rs2227307 | IL-8 | 74606669 | T | 0.4788 | 0.5 | G | 0.6144 | 0.9187 | 0.6408 | 1.317 | 1 | 0.9257 | -0.00293707 | 0.4895 |
| 4 | rs2227545 | IL-8 | 74608727 | C | 0.07563 | 0.08824 | A | 0.6215 | 0.8455 | 0.4383 | 1.631 | 1 | 0.9257 | -0.00327016 | 0.08193 |
| 4 | rs58478511 | IL-8 | 74610033 | A | 0.2348 | 0.2261 | G | 0.8259 | 1.05 | 0.6804 | 1.621 | 1 | 0.9281 | -0.004256 | 0.2304 |
| 5 | rs2546890 | IL-12B | 158759900 | A | 0.3051 | 0.3193 | G | 0.7294 | 0.9358 | 0.6345 | 1.38 | 1 | 0.9257 | -0.00402212 | 0.3122 |
| 5 | rs3212227 | IL-12B | 158742950 | G | 0.4412 | 0.411 | T | 0.4872 | 1.131 | 0.7859 | 1.629 | 1 | 0.9257 | -0.00260248 | 0.4262 |
| 5 | rs2243250 | IL-4 | 132009154 | C | 0.2353 | 0.2437 | T | 0.831 | 0.9549 | 0.6268 | 1.455 | 1 | 0.9281 | -0.00446726 | 0.2395 |
| 5 | rs2243255 | IL-4 | 132011737 | A | 0.1096 | 0.1681 | G | 0.06764 | 0.6093 | 0.3542 | 1.048 | 1 | 0.5496 | 0.0100856 | 0.1388 |
| 5 | rs2243256 | IL-4 | 132011753 | DEL | 0.1303 | 0.09664 | T | 0.2523 | 1.4 | 0.79 | 2.481 | 1 | 0.8752 | 0.00158722 | 0.1134 |
| 5 | rs2243258 | IL-4 | 132012110 | T | 0.07563 | 0.06723 | C | 0.7265 | 1.135 | 0.5644 | 2.283 | 1 | 0.9257 | -0.0036442 | 0.07143 |
| 5 | rs2243261 | IL-4 | 132012806 | T | 0.07983 | 0.05462 | G | 0.2799 | 1.502 | 0.7239 | 3.115 | 1 | 0.8752 | 0.00115422 | 0.06695 |
| 5 | rs2243268 | IL-4 | 132013963 | C | 0.4202 | 0.3739 | A | 0.3049 | 1.213 | 0.8399 | 1.752 | 1 | 0.8752 | 0.000652226 | 0.3971 |
| 5 | rs2243279 | IL-4 | 132016227 | A | 0.06723 | 0.06723 | G | 0.9276 | 1 | 0.488 | 2.049 | 1 | 0.9519 | -0.0039319 | 0.06723 |
| **5** | **rs2243283** | **IL-4** | **132016593** | **G** | **0.275** | **0.1875** | **C** | **0.04006** | **1.644** | **1.031** | **2.621** | **1** | **0.4339** | **0.0158649** | **0.2304** |
| 5 | rs73269366 | IL-4 | 132018749 | T | 0.01681 | 0.03361 | C | 0.2612 | 0.4915 | 0.146 | 1.655 | 1 | 0.8752 | 0.00163907 | 0.02521 |
| 5 | rs734244 | IL-4 | 132010726 | C | 0.4703 | 0.5042 | T | 0.4362 | 0.8732 | 0.609 | 1.252 | 1 | 0.9257 | -0.00300466 | 0.4873 |
| 5 | rs9282745 | IL-4 | 132014000 | A | 0.02119 | 0.04202 | T | 0.2477 | 0.4935 | 0.1661 | 1.466 | 1 | 0.8752 | 0.00914955 | 0.03165 |
| **6** | **rs1233330** | **HLA-G** | **29799103** | **A** | **0.1008** | **0.1597** | **G** | **0.05823** | **0.5903** | **0.3419** | **1.019** | **1** | **0.4339** | **0.0142926** | **0.1055** |
| **6** | **rs1233330** | **HLA-G** | **29799103** | **A** | **0.07563** | **0.1356** | **G** | **0.03048** | **0.5216** | **0.284** | **0.9581** | **1** | **0.5407** | **0.00914955** | **0.1303** |
| 6 | rs12662618 | HLA-G | 29800211 | C | 0.08475 | 0.09664 | T | 0.6921 | 0.8655 | 0.4618 | 1.622 | 1 | 0.9257 | -0.00340373 | 0.09072 |
| 6 | rs141206123 | HLA-G | 29799849 | C | 0.07983 | 0.07983 | DEL | 0.933 | 1 | 0.5154 | 1.94 | 1 | 0.9519 | -0.00459676 | 0.07983 |
| 6 | rs142798055 | HLA-G | 29793404 | DEL | 0.09709 | 0.1179 | TCT | 0.4807 | 0.8043 | 0.4318 | 1.498 | 1 | 0.9257 | -0.00293754 | 0.1077 |
| 6 | rs1610696 | HLA-G | 29798803 | G | 0.1441 | 0.1538 | C | 0.7472 | 0.9257 | 0.557 | 1.539 | 1 | 0.9257 | -0.00532233 | 0.1489 |
| 6 | rs1632932 | HLA-G | 29798039 | A | 0.3551 | 0.2929 | G | 0.1896 | 1.329 | 0.878 | 2.013 | 1 | 0.8752 | 0.005317 | 0.3252 |
| 6 | rs17875389 | HLA-G | 29794484 | G | 0.05085 | 0.03361 | A | 0.3122 | 1.54 | 0.6179 | 3.839 | 1 | 0.8752 | -0.00126392 | 0.04219 |
| 6 | rs2517898 | HLA-G | 29799746 | G | 0.2941 | 0.339 | C | 0.301 | 0.8125 | 0.5513 | 1.197 | 1 | 0.8752 | 0.000642655 | 0.3165 |
| **6** | **rs9380142** | **HLA-G** | **29798794** | **G** | **0.3686** | **0.2415** | **A** | **0.002777** | **1.834** | **1.231** | **2.731** | **0.1805** | **0.1805** | **0.030427** | **0.3051** |
| 6 | rs1800629 | TNF-A | 31543031 | A | 0.1092 | 0.09664 | G | 0.6549 | 1.146 | 0.6341 | 2.073 | 1 | 0.9257 | -0.00307857 | 0.1029 |
| **6** | **rs1800630** | **TNF-A** | **31542476** | **A** | **0.1555** | **0.09244** | **C** | **0.03809** | **1.807** | **1.031** | **3.169** | **1** | **0.4339** | **0.0141093** | **0.1239** |
| 7 | rs1818879 | IL-6 | 22772727 | A | 0.1325 | 0.1282 | G | 0.8919 | 1.038 | 0.6062 | 1.779 | 1 | 0.9519 | -0.00423326 | 0.1303 |
| 7 | rs2066992 | IL-6 | 22768249 | T | 0.06303 | 0.07627 | G | 0.5321 | 0.8146 | 0.4004 | 1.657 | 1 | 0.9257 | -0.00285209 | 0.06962 |
| 7 | rs2069830 | IL-6 | 22767137 | T | 0.09244 | 0.07143 | C | 0.4102 | 1.324 | 0.6843 | 2.562 | 1 | 0.9257 | -0.00185698 | 0.08193 |
| 7 | rs2069834 | IL-6 | 22767828 | T | 0.04202 | 0.07983 | C | 0.08883 | 0.5055 | 0.2299 | 1.112 | 1 | 0.6415 | 0.00791584 | 0.06092 |
| 7 | rs2069837 | IL-6 | 22768027 | G | 0.105 | 0.1429 | A | 0.2148 | 0.7042 | 0.4059 | 1.222 | 1 | 0.8752 | 0.00263809 | 0.1239 |
| 7 | rs2069843 | IL-6 | 22769994 | A | 0.1639 | 0.1975 | G | 0.344 | 0.7964 | 0.4984 | 1.273 | 1 | 0.8752 | -0.000431748 | 0.1807 |
| 7 | rs2069845 | IL-6 | 22770149 | G | 0.3193 | 0.3613 | A | 0.3356 | 0.8292 | 0.5672 | 1.212 | 1 | 0.8752 | -0.000164277 | 0.3403 |
| 7 | rs2069855 | IL-6 | 22772624 | C | 0.02941 | 0.03782 | T | 0.6234 | 0.771 | 0.2824 | 2.105 | 1 | 0.9257 | -0.0035445 | 0.03361 |
| 7 | rs62449495 | IL-6 | 22764338 | A | 0.07983 | 0.05462 | G | 0.2799 | 1.502 | 0.7239 | 3.115 | 1 | 0.8752 | 0.00115422 | 0.06723 |
| 12 | rs1861493 | IFN-ϒ | 68551196 | G | 0.05882 | 0.05042 | A | 0.6933 | 1.177 | 0.5327 | 2.601 | 1 | 0.9257 | -0.003994 | 0.05462 |
| 12 | rs2069705 | IFN-ϒ | 68555011 | G | 0.4286 | 0.3824 | A | 0.3066 | 1.212 | 0.8399 | 1.748 | 1 | 0.8752 | 0.000294768 | 0.4055 |
| 12 | rs2069713 | IFN-ϒ | 68552476 | C | 0.07563 | 0.06303 | T | 0.5948 | 1.216 | 0.598 | 2.474 | 1 | 0.9257 | -0.00295927 | 0.06933 |
| 12 | rs2069720 | IFN-ϒ | 68549710 | T | 0.1017 | 0.1261 | C | 0.4295 | 0.7849 | 0.444 | 1.388 | 1 | 0.9257 | -0.00199975 | 0.1139 |
| 12 | rs2069722 | IFN-ϒ | 68548953 | A | 0.02809 | 0.04717 | G | 0.364 | 0.5838 | 0.1958 | 1.741 | 1 | 0.8764 | -0.000786883 | 0.03846 |
| 12 | rs2069723 | IFN-ϒ | 68548594 | C | 0.008475 | 0.01261 | T | 0.8424 | 0.6695 | 0.1109 | 4.044 | 1 | 0.9281 | -0.00338787 | 0.01055 |
| 12 | rs2069728 | IFN-ϒ | 68547784 | T | 0.2269 | 0.2479 | C | 0.5924 | 0.8904 | 0.5835 | 1.359 | 1 | 0.9257 | -0.00297077 | 0.2374 |
| 12 | rs2430561 | IFN-ϒ | 68552522 | A | 0.1767 | 0.1765 | T | 0.9519 | 1.002 | 0.6235 | 1.61 | 1 | 0.9519 | -0.00450227 | 0.1766 |
| 12 | rs78554979 | IFN-ϒ | 68554636 | C | 0.0678 | 0.07143 | T | 0.9294 | 0.9455 | 0.4659 | 1.919 | 1 | 0.9519 | -0.0041117 | 0.06962 |
| 16 | rs1424241 | TXNL4B | 72078907 | A | 0.1176 | 0.1154 | G | 0.9432 | 1.022 | 0.5825 | 1.794 | 1 | 0.9519 | -0.00456464 | 0.1165 |
| 16 | rs7185840 | HPR | 72102112 | A | 0.1597 | 0.1765 | G | 0.6267 | 0.8867 | 0.5481 | 1.434 | 1 | 0.9257 | -0.00427472 | 0.1681 |
| 16 | rs8062041 | HP | 72088964 | C | 0.4496 | 0.4831 | T | 0.4912 | 0.8741 | 0.6092 | 1.254 | 1 | 0.9257 | -0.00181108 | 0.4662 |
| 16 | rs1801275 | IL-4R | 27374400 | A | 0.2034 | 0.2203 | G | 0.6545 | 0.9034 | 0.5808 | 1.405 | 1 | 0.9257 | -0.00366904 | 0.2119 |
| 19 | rs1736936 | HLAG | 29794317 | A | 0.4286 | 0.416 | G | 0.7817 | 1.053 | 0.7319 | 1.515 | 1 | 0.9257 | -0.0040236 | 0.4223 |
| 19 | rs11575934 | IL-12RB1 | 18186618 | C | 0.06723 | 0.08974 | T | 0.3501 | 0.731 | 0.3715 | 1.439 | 1 | 0.8752 | -0.000385708 | 0.07806 |
| 22 | rs136177 | APOL1 | 36661842 | G | 0.1398 | 0.1092 | A | 0.2993 | 1.326 | 0.7657 | 2.295 | 1 | 0.8752 | 0.000494383 | 0.1245 |
| 22 | rs71785313 | APOL1 | 36662046 | T | 0.08051 | 0.08621 | A | 0.8035 | 0.9281 | 0.4817 | 1.788 | 1 | 0.9281 | -0.00394606 | 0.08333 |
| 22 | rs73885316 | APOL1 | 36661674 | A | 0.04202 | 0.0339 | C | 0.7255 | 1.25 | 0.4846 | 3.225 | 1 | 0.9257 | -0.00318007 | 0.03797 |
| 22 | rs73885319 | APOL1 | 36661906 | G | 0.07563 | 0.1008 | A | 0.3386 | 0.7295 | 0.3849 | 1.383 | 1 | 0.8752 | 0.000139201 | 0.08824 |
| **22** | **rs34383331** | **MIF** | **24238079** | **A** | **0.2395** | **0.1597** | **T** | **0.03006** | **1.657** | **1.049** | **2.618** | **1** | **0.4339** | **0.0150296** | **0.1996** |
| 22 | rs35235644 | MIF | 24237822 | C | 0.09244 | 0.08403 | G | 0.7501 | 1.11 | 0.5889 | 2.093 | 1 | 0.9257 | -0.0040495 | 0.08824 |
| 22 | rs36086171 | MIF | 24235455 | G | 0.3077 | 0.3277 | A | 0.6574 | 0.9117 | 0.6187 | 1.343 | 1 | 0.9257 | -0.00510933 | 0.3178 |
| **22** | **rs9282783** | **MIF** | **24236359** | **G** | **0.08898** | **0.04202** | **C** | **0.03328** | **2.227** | **1.025** | **4.837** | **1** | **0.4339** | **0.012872** | **0.0654** |

*Abbreviations: CHR = Chromosome, SNP = SNP ID, BP = Physical position (base-pair), A1 = Minor allele (based on whole sample), F_A = Frequency of allele 1 in cases, F_U = Frequency of allele 1 in controls, A2 = Major allele, P = p-value for this test, OR = Estimated odds ratio (for A1, i.e. A2 is reference), BONF = Bonferroni single-step adjusted p-values, FDR_BH = Benjamini & Hochberg (1995) step-up FDR control, FST = Fixation index, and MAF = Minor allele frequency. The level of significance is 0.05.
