# Supplementary material for: No evidence for association between APOL1 kidney disease risk alleles and Human African Trypanosomiasis in two Ugandan populations
Source: PLoS Negl Trop Dis. 2018 Feb 22;12(2):e0006300. doi: 10.1371/journal.pntd.0006300 (PMC5844566; doi:10.1371/journal.pntd.0006300)
Supplement: S3 Table — (DOCX) [file pntd.0006300.s003.docx]

**S3 Table: Association results of 65 SNPs with Chronic HAT**

| **CHR** | **SNP** | **GENE** | **BP** | **A1** | **F_A** | **F_U** | **A2** | **P** | **OR** | **L95** | **U95** | **BONF** | **FDR_BH** | **FST** | **MAF** |
| --- | --- | --- | --- | --- | --- | --- | --- | --- | --- | --- | --- | --- | --- | --- | --- |
| **1** | **rs1061170** | **CFH** | **196659237** | **C** | **0.4091** | **0.5248** | **T** | **0.0188** | **0.627** | **0.4221** | **0.9313** | **1** | **0.6107** | **0.022134** | **0.4675** |
| 1 | rs1800872 | IL-10 | 206946407 | T | 0.4457 | 0.4646 | G | 0.7198 | 0.9263 | 0.619 | 1.386 | 1 | 0.8928 | -0.00550129 | 0.4555 |
| 2 | rs1143629 | IL-1B | 113593518 | A | 0.3265 | 0.35 | G | 0.6338 | 0.9004 | 0.5936 | 1.366 | 1 | 0.8653 | -0.00378649 | 0.3384 |
| 4 | rs114259658 | IL-8 | 74605639 | A | 0.05556 | 0.02427 | T | 0.1006 | 2.365 | 0.8065 | 6.934 | 1 | 0.6902 | 0.00808187 | 0.0396 |
| 4 | rs2227307 | IL-8 | 74606669 | T | 0.3333 | 0.4078 | G | 0.1334 | 0.7262 | 0.4824 | 1.093 | 1 | 0.7228 | 0.00717949 | 0.3719 |
| 4 | rs2227545 | IL-8 | 74608727 | C | 0.1111 | 0.07767 | A | 0.2716 | 1.484 | 0.7552 | 2.918 | 1 | 0.8653 | 0.00152592 | 0.09406 |
| 4 | rs58478511 | IL-8 | 74610033 | A | 0.4105 | 0.3788 | G | 0.5009 | 1.142 | 0.7599 | 1.717 | 1 | 0.8653 | -0.00441686 | 0.3943 |
| 5 | rs2243250 | IL-4 | 132009154 | C | 0.2323 | 0.1942 | T | 0.3639 | 1.256 | 0.7791 | 2.024 | 1 | 0.8653 | -0.000908447 | 0.2129 |
| 5 | rs734244 | IL-4 | 132010726 | T | 0.4242 | 0.4417 | C | 0.7259 | 0.9312 | 0.6281 | 1.381 | 1 | 0.8928 | -0.00458497 | 0.4332 |
| 5 | rs2243256 | IL-4 | 132011753 | DEL | 0.05051 | 0.03398 | T | 0.3937 | 1.512 | 0.564 | 4.054 | 1 | 0.8653 | -0.00137587 | 0.04208 |
| 5 | rs2243258 | IL-4 | 132012110 | T | 0.07071 | 0.1262 | C | 0.05637 | 0.5268 | 0.2665 | 1.041 | 1 | 0.6107 | 0.0116914 | 0.09901 |
| 5 | rs2243261 | IL-4 | 132012806 | T | 0.2424 | 0.2039 | G | 0.3718 | 1.25 | 0.7813 | 1.998 | 1 | 0.8653 | -0.000686061 | 0.2228 |
| 5 | rs9282745 | IL-4 | 132014000 | A | 0.03061 | 0.02913 | T | 0.8859 | 1.053 | 0.3337 | 3.321 | 1 | 0.914 | -0.00483513 | 0.02985 |
| 5 | rs2243270 | IL-4 | 132014109 | A | 0.1939 | 0.1942 | G | 0.9498 | 0.9981 | 0.6087 | 1.637 | 1 | 0.9498 | -0.00493724 | 0.194 |
| 5 | rs2243279 | IL-4 | 132016227 | A | 0.06566 | 0.1019 | G | 0.1819 | 0.619 | 0.301 | 1.273 | 1 | 0.7884 | 0.00338443 | 0.08416 |
| 5 | rs2243283 | IL-4 | 132016593 | G | 0.1986 | 0.2216 | C | 0.6331 | 0.8707 | 0.5073 | 1.494 | 1 | 0.8653 | -0.00612429 | 0.2112 |
| 5 | rs73269366 | IL-4 | 132018749 | T | 0.0101 | 0.01942 | C | 0.5634 | 0.5153 | 0.09332 | 2.845 | 1 | 0.8653 | -0.00194264 | 0.01485 |
| 5 | rs3212227 | IL-12B | 158742950 | G | 0.3939 | 0.3627 | T | 0.505 | 1.142 | 0.7628 | 1.709 | 1 | 0.8653 | -0.00318415 | 0.3781 |
| 5 | rs2546890 | IL-12B | 158759900 | A | 0.4545 | 0.4596 | G | 0.9201 | 0.9799 | 0.6598 | 1.455 | 1 | 0.9344 | -0.0051162 | 0.4571 |
| 6 | rs142798055 | HLA-G | 29793404 | DEL | 0.2065 | 0.137 | TCT | 0.09539 | 1.64 | 0.9075 | 2.963 | 1 | 0.6902 | 0.0110264 | 0.1758 |
| 6 | rs17875389 | HLA-G | 29794484 | G | 0.06566 | 0.04902 | A | 0.4582 | 1.363 | 0.5835 | 3.185 | 1 | 0.8653 | -0.0026029 | 0.05721 |
| 6 | rs17179108 | HLA-G | 29798642 | T | 0.1162 | 0.1408 | C | 0.5074 | 0.8022 | 0.4465 | 1.441 | 1 | 0.8653 | -0.00198705 | 0.1287 |
| 6 | rs9380142 | HLA-G | 29798794 | G | 0.2419 | 0.2157 | A | 0.5085 | 1.161 | 0.723 | 1.863 | 1 | 0.8653 | -0.00742882 | 0.2282 |
| 6 | rs1610696 | HLA-G | 29798803 | G | 0.2368 | 0.17 | C | 0.09037 | 1.515 | 0.9209 | 2.493 | 1 | 0.6902 | 0.00734915 | 0.2026 |
| **6** | **rs1233330** | **HLA-G** | **29799103** | **A** | **0.07576** | **0.1359** | **G** | **0.04453** | **0.5211** | **0.2693** | **1.008** | **1** | **0.6107** | **0.0120013** | **0.1064** |
| 6 | rs1233330 | HLA-G | 29799103 | A | 0.07576 | 0.1068 | G | 0.2659 | 0.6855 | 0.3447 | 1.363 | 1 | 0.8653 | 0.000130209 | 0.09158 |
| 6 | rs141206123 | HLA-G | 29799849 | C | 0.0202 | 0.03883 | DEL | 0.3165 | 0.5103 | 0.1512 | 1.722 | 1 | 0.8653 | 0.00120087 | 0.0297 |
| 6 | rs2517897 | HLA-G | 29800101 | A | 0.2727 | 0.267 | C | 0.8669 | 1.03 | 0.6634 | 1.598 | 1 | 0.9089 | -0.0075944 | 0.2698 |
| 6 | rs12662618 | HLA-G | 29800211 | C | 0.2121 | 0.1814 | T | 0.4168 | 1.215 | 0.7423 | 1.989 | 1 | 0.8653 | -0.00141927 | 0.1965 |
| 6 | rs1059564 | HLA-A | 29911930 | T | 0 | 0.00495 | C | 0.7437 | 0 | 0 | nan | 1 | 0.8928 | -0.000252777 | 0.002538 |
| 6 | rs1800630 | TNF-A | 31542476 | A | 0.09596 | 0.08738 | C | 0.798 | 1.109 | 0.5637 | 2.18 | 1 | 0.8928 | -0.00464719 | 0.09158 |
| 6 | rs1800629 | TNF-A | 31543031 | A | 0.08586 | 0.05825 | G | 0.2934 | 1.518 | 0.7057 | 3.267 | 1 | 0.8653 | 0.00112916 | 0.07178 |
| 7 | rs62449495 | IL-6 | 22764338 | A | 0.05051 | 0.03883 | G | 0.5533 | 1.316 | 0.5087 | 3.407 | 1 | 0.8653 | -0.00373978 | 0.04455 |
| 7 | rs2069830 | IL-6 | 22767137 | T | 0.005051 | 0.009709 | C | 0.8084 | 0.5178 | 0.04658 | 5.756 | 1 | 0.8928 | -0.00348262 | 0.007426 |
| 7 | rs2069834 | IL-6 | 22767828 | T | 0.0202 | 0.01456 | C | 0.5845 | 1.395 | 0.3083 | 6.315 | 1 | 0.8653 | 0.0184311 | 0.01733 |
| 7 | rs2069837 | IL-6 | 22768027 | G | 0.1212 | 0.1311 | A | 0.8241 | 0.9144 | 0.5079 | 1.647 | 1 | 0.8928 | -0.00450927 | 0.1262 |
| 7 | rs1474347 | IL-6 | 22768124 | C | 0.1111 | 0.1019 | A | 0.8108 | 1.101 | 0.585 | 2.073 | 1 | 0.8928 | -0.00500229 | 0.1064 |
| 7 | rs2066992 | IL-6 | 22768249 | T | 0.1211 | 0.1716 | G | 0.179 | 0.665 | 0.3769 | 1.173 | 1 | 0.7884 | 0.00531762 | 0.1472 |
| **7** | **rs2069843** | **IL-6** | **22769994** | **A** | **0.1465** | **0.07767** | **G** | **0.03313** | **2.038** | **1.07** | **3.882** | **1** | **0.6107** | **0.0184311** | **0.1114** |
| 7 | rs2069845 | IL-6 | 22770149 | G | 0.303 | 0.2379 | A | 0.1315 | 1.393 | 0.896 | 2.166 | 1 | 0.7228 | 0.00576148 | 0.2698 |
| 7 | rs2069855 | IL-6 | 22772624 | C | 0.005051 | 0.01456 | T | 0.4934 | 0.3435 | 0.03543 | 3.33 | 1 | 0.8653 | 0.00576148 | 0.009901 |
| 7 | rs1818879 | IL-6 | 22772727 | A | 0.2576 | 0.2822 | G | 0.614 | 0.8826 | 0.5673 | 1.373 | 1 | 0.8653 | -0.0033028 | 0.27 |
| 12 | rs2069728 | IFN-ϒ | 68547784 | T | 0.3081 | 0.3301 | C | 0.6321 | 0.9036 | 0.5945 | 1.374 | 1 | 0.8653 | -0.00426685 | 0.3193 |
| 12 | rs2069723 | IFN-ϒ | 68548594 | C | 0.01562 | 0 | T | 0.05568 | NA | NA | NA | 1 | 0.6107 | 0.00787836 | 0.007538 |
| 12 | rs2069722 | IFN-ϒ | 68548953 | A | 0.075 | 0.05263 | G | 0.4018 | 1.459 | 0.5752 | 3.703 | 1 | 0.8653 | -0.00282761 | 0.06129 |
| 12 | rs2069720 | IFN-ϒ | 68549710 | T | 0.1263 | 0.1456 | C | 0.6144 | 0.8478 | 0.4791 | 1.5 | 1 | 0.8653 | -0.00302951 | 0.1361 |
| 12 | rs1861493 | IFN-ϒ | 68551196 | G | 0.06566 | 0.07767 | A | 0.6336 | 0.8345 | 0.3905 | 1.783 | 1 | 0.8653 | -0.00389626 | 0.07178 |
| 12 | rs2069713 | IFN-ϒ | 68552476 | C | 0.0101 | 0.009709 | T | 0.8117 | 1.041 | 0.1452 | 7.461 | 1 | 0.8928 | -0.00494404 | 0.009901 |
| 12 | rs2430561 | IFN-ϒ | 68552522 | A | 0.1429 | 0.1068 | T | 0.2598 | 1.394 | 0.7679 | 2.53 | 1 | 0.8653 | 0.000765078 | 0.1244 |
| **12** | **rs78554979** | **IFN-ϒ** | **68554636** | **C** | **0.05102** | **0.01456** | **T** | **0.03478** | **3.638** | **0.9861** | **13.42** | **1** | **0.6107** | **0.0163105** | **0.03234** |
| 12 | rs2069705 | IFN-ϒ | 68555011 | G | 0.3131 | 0.2913 | A | 0.6268 | 1.109 | 0.7253 | 1.697 | 1 | 0.8653 | -0.00439728 | 0.302 |
| 16 | rs1801275 | IL-4R | 27374400 | A | 0.1414 | 0.1553 | G | 0.7283 | 0.8956 | 0.517 | 1.551 | 1 | 0.8928 | -0.00453209 | 0.1485 |
| 16 | rs7185840 | HPR | 72102112 | A | 0.1869 | 0.2524 | G | 0.1062 | 0.6806 | 0.4229 | 1.095 | 1 | 0.6902 | 0.00749627 | 0.2203 |
| 16 | rs2021171 | HPR | 72110541 | A | 0.2172 | 0.1961 | G | 0.5809 | 1.137 | 0.7015 | 1.844 | 1 | 0.8653 | -0.00311486 | 0.2065 |
| 19 | rs11575934 | IL-12RB1 | 18186618 | C | 0.1818 | 0.2206 | T | 0.3532 | 0.7852 | 0.4811 | 1.281 | 1 | 0.8653 | 0.00000416 | 0.2015 |
| 19 | rs1736936 | HLA-G | 29794317 | A | 0.5303 | 0.4554 | G | 0.1483 | 1.35 | 0.9111 | 2 | 1 | 0.7416 | 0.00623214 | 0.4925 |
| 22 | rs36086171 | MIF | 24235455 | G | 0.2576 | 0.297 | A | 0.4043 | 0.8211 | 0.5295 | 1.273 | 1 | 0.8653 | -0.00273115 | 0.2775 |
| 22 | rs9282783 | MIF | 24236359 | G | 0.08081 | 0.06796 | C | 0.639 | 1.206 | 0.5721 | 2.541 | 1 | 0.8653 | -0.00519226 | 0.07426 |
| 22 | rs35235644 | MIF | 24237822 | C | 0.08081 | 0.07282 | G | 0.7817 | 1.119 | 0.5378 | 2.33 | 1 | 0.8928 | -0.00518057 | 0.07673 |
| 22 | rs34383331 | MIF | 24238079 | A | 0.2879 | 0.301 | T | 0.7857 | 0.9389 | 0.6119 | 1.441 | 1 | 0.8928 | -0.00452386 | 0.2946 |
| 22 | rs136174 | APOL1 | 36661536 | C | 0.0404 | 0.02451 | A | 0.3349 | 1.676 | 0.5387 | 5.213 | 1 | 0.8653 | -0.000799842 | 0.03234 |
| 22 | rs73885316 | APOL1 | 36661674 | A | 0.0303 | 0.02451 | C | 0.656 | 1.244 | 0.3734 | 4.143 | 1 | 0.8702 | -0.00424983 | 0.02736 |
| 22 | rs136177 | APOL1 | 36661842 | G | 0.07576 | 0.04902 | A | 0.2608 | 1.59 | 0.6967 | 3.629 | 1 | 0.8653 | 0.00103504 | 0.06219 |
| 22 | rs73885319 | APOL1 | 36661906 | G | 0.01515 | 0.01456 | A | 0.8428 | 1.041 | 0.2076 | 5.22 | 1 | 0.898 | -0.00491464 | 0.06281 |
| 22 | rs71785313 | APOL1 | 36662046 | T | 0.05556 | 0.07 | A | 0.6115 | 0.7815 | 0.3458 | 1.766 | 1 | 0.8653 | -0.00295232 | 0.06281 |

*Abbreviations: CHR = Chromosome, SNP = SNP ID, BP = Physical position (base-pair), A1 = Minor allele (based on whole sample), F_A = Frequency of allele 1 in cases, F_U = Frequency of allele 1 in controls, A2 = Major allele, P = p-value for this test, OR = Estimated odds ratio (for A1, i.e. A2 is reference), BONF = Bonferroni single-step adjusted p-values, FDR_BH = Benjamini & Hochberg (1995) step-up FDR control, FST = Fixation index, and MAF = Minor allele frequency. The level of significance is 0.05.
