# Supplementary figures and images for: No evidence for association between APOL1 kidney disease risk alleles and Human African Trypanosomiasis in two Ugandan populations
Source: PLoS Negl Trop Dis. 2018 Feb 22;12(2):e0006300. doi: 10.1371/journal.pntd.0006300 (PMC5844566; doi:10.1371/journal.pntd.0006300)

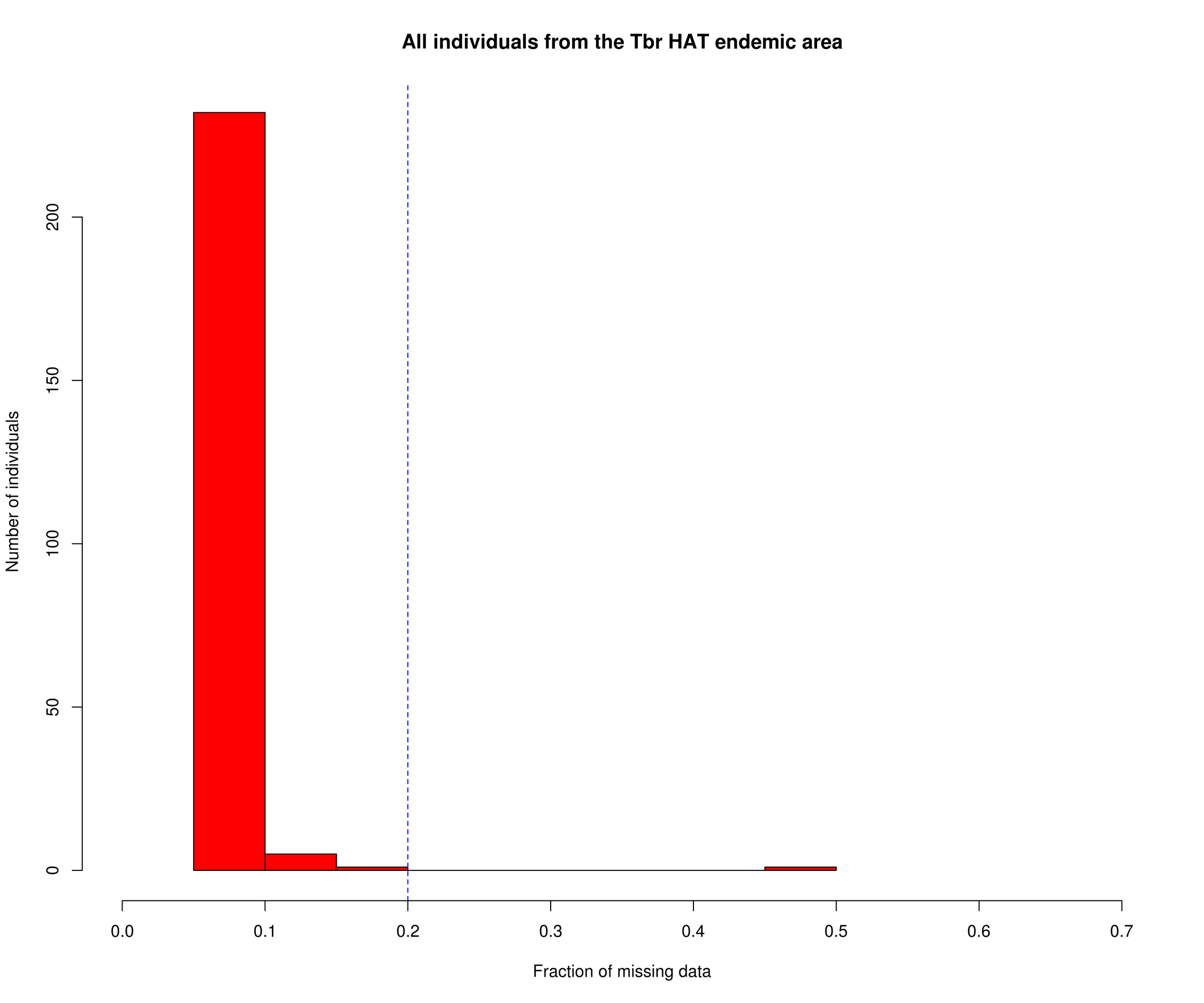

Supplement: S1 Fig — (TIF) [file pntd.0006300.s008.tif]

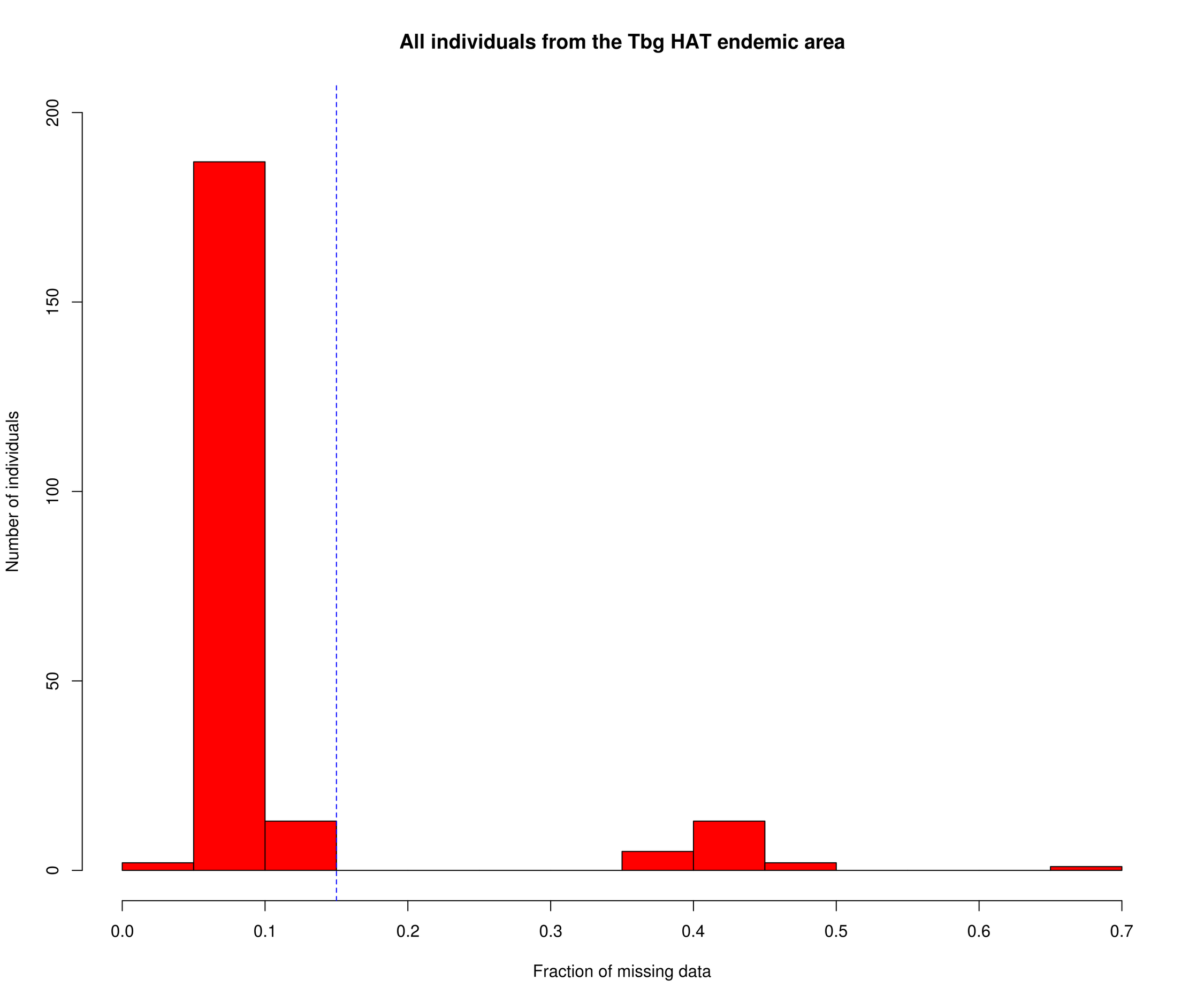

Supplement: S2 Fig — (TIF) [file pntd.0006300.s009.tif]

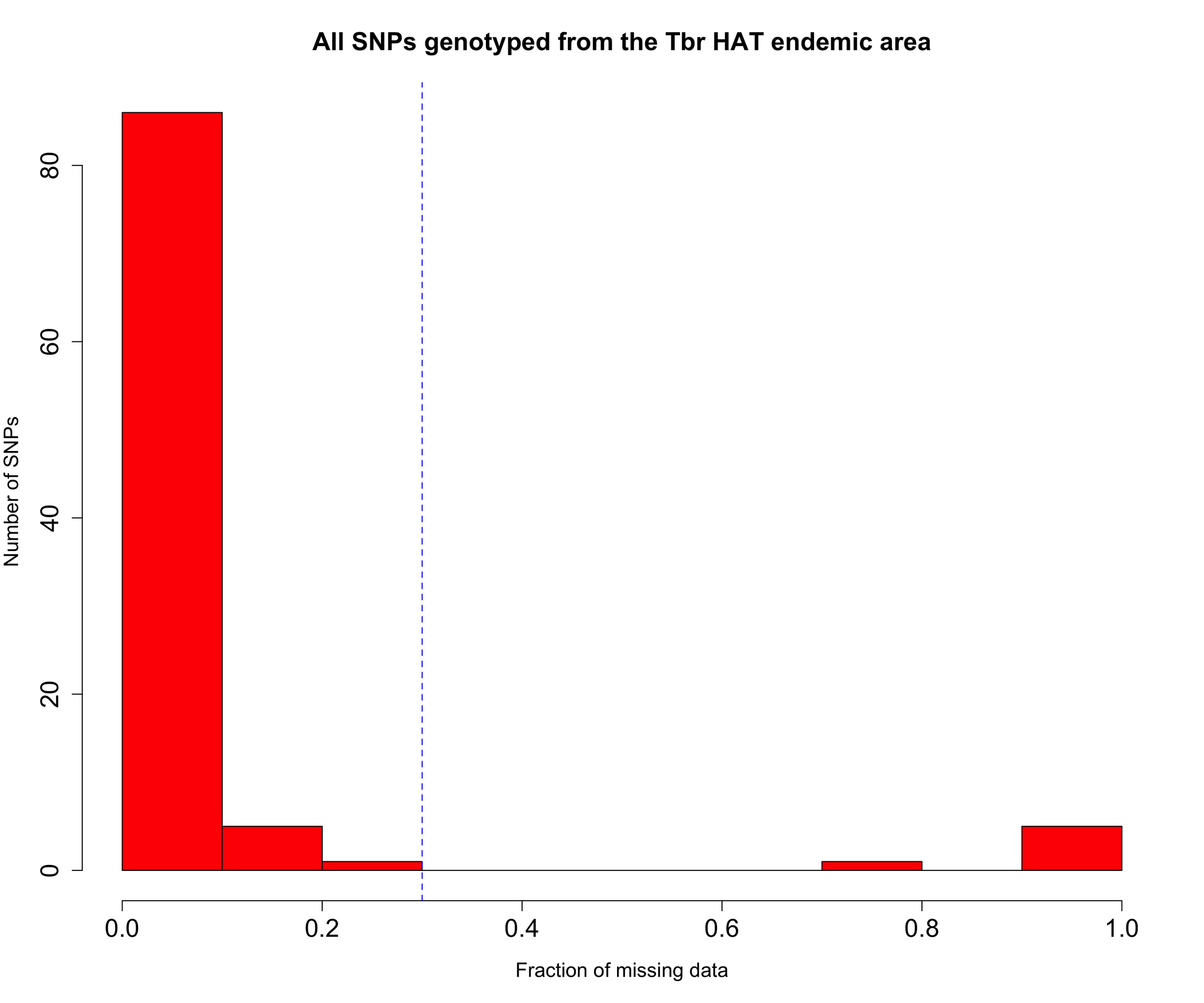

Supplement: S3 Fig — (TIF) [file pntd.0006300.s010.tif]

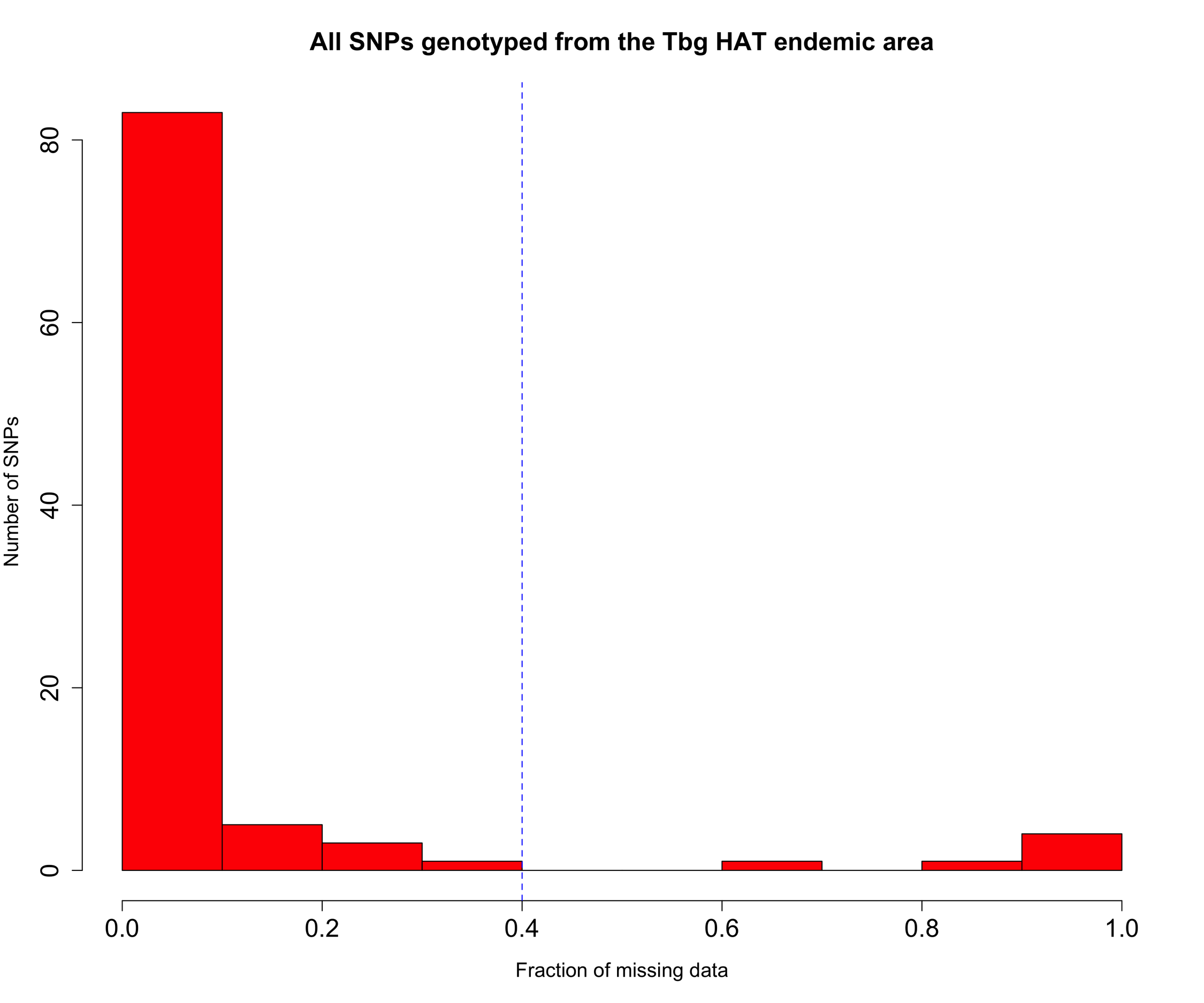

Supplement: S4 Fig — (TIF) [file pntd.0006300.s011.tif]

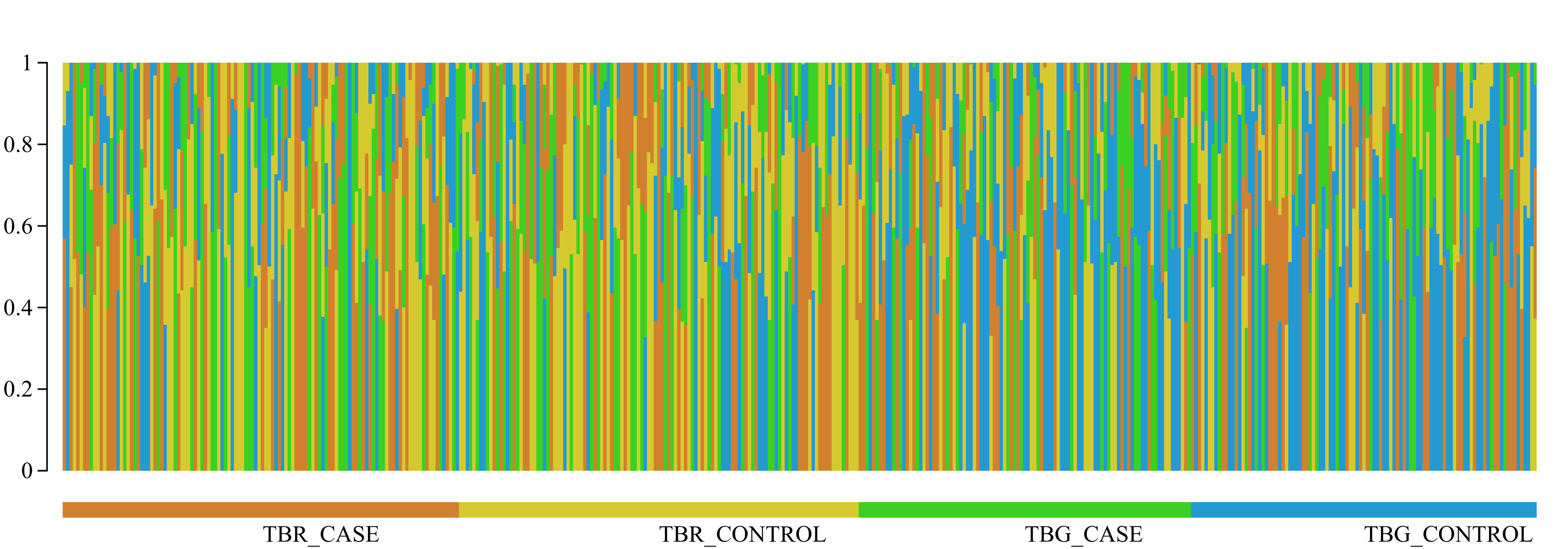

Supplement: S5 Fig — (TIF) [file pntd.0006300.s012.tif]
